# Impacts of the Internet on Health Inequality and Healthcare Access: A Cross-Country Study

**DOI:** 10.3389/fpubh.2022.935608

**Published:** 2022-06-09

**Authors:** Jiajie Yu, Shuang Meng

**Affiliations:** ^1^Business School, Beijing Normal University, Beijing, China; ^2^School of International Trade and Economics, Central University of Finance and Economics, Beijing, China

**Keywords:** internet, health inequality, healthcare access, income inequality, cross-country

## Abstract

Access to information and resources through the Internet has become an increasingly critical aspect of contemporary life. Based on the WHO Health Equity Assessment Toolkit (HEAT) and cross-country panel data, this paper investigates the effect of Internet access on health inequality across different income groups. The results indicate that access to the Internet significantly improves the average health condition and alleviates health inequality. In addition, employing cross-country data from the Global Burden of Disease (GBD) database, this paper further examines the social and economic determinants of access to healthcare. Specifically, it is found that Internet access significantly facilitates healthcare access and mitigates the negative impact of income inequality on healthcare access. Considered together, these findings shed light on the importance of the Internet in reducing health inequality and improving healthcare access.

## Introduction

Reducing health inequality and improving healthcare access are of vital importance in the field of public health, both academically and practically speaking. On the one hand, health inequality, which generically refers to systematic differences in the health status or in the distribution of health resources between different population groups, has substantial social and economic impacts on individuals and societies (1, 2). On the other hand, addressing health literacy, enhancing the physician-patient relationship, and identifying cost-effective resources are essential means of promoting access to healthcare, which can translate into significant public health gains. One of the most prominent characteristics of current public health services is that healthcare has been undergoing a major digital transformation due to the extensive use of information and communication technologies (ICTs). In particular, the widespread diffusion of the Internet has enabled better access to health information and resources, generating both distributional and aggregate effects on health outcomes (3–6). For instance, web-based medical service (WBMS), which is defined broadly as a cooperative relationship between Internet technology and medical service, has been considered one of the most innovative health services in the digital age (7, 8). The use of WBMS, such as telehealth, eHealth, and mHealth, has greatly facilitated the distribution of health-related information and resources via the Internet across different social groups. Therefore, the increasing prevalence of the Internet has empowered people worldwide, in general and especially those in need, to access healthcare at the point of care or remotely. Healthcare providers have been using the Internet to enhance their skills and knowledge and, more importantly, to provide patients with assistance and guidance if necessary (9, 10). Despite the important role that Internet access plays in public health, relatively few studies have systematically examined the distributional and aggregate effects of the Internet on health outcomes.

To address this critical gap in the literature, this paper conducts a cross-country study to investigate the impacts of the Internet on health inequality and healthcare access. First, this paper quantifies the effect of Internet access on health inequality across different income groups. It is found that increased access to the Internet significantly reduces health inequality and improves the overall health condition. The gap in health status between the poor and the rich would be reduced if the Internet became more accessible. The pattern persists when we control for a wide range of variables that potentially influence health inequality in the estimation. Second, this paper explores the social and economic determinants of healthcare access. Specifically, among all of the factors included in the estimation, we focus on the impacts of Internet access, income inequality, and their interaction. It is shown that an improvement of Internet access facilitates healthcare access, while an increase in income inequality impedes access to healthcare. Moreover, Internet access significantly mitigates the negative impact of income inequality on healthcare access.

The main contributions of this paper are threefold. First, this paper sheds light on the relationship between the Internet and major health outcomes. We show that Internet access plays an important role in influencing health inequality and healthcare access. Our findings suggest that increasing Internet penetration and reducing barriers to accessing health information could be promising public health interventions. Second, this paper makes a novel contribution by investigating how Internet affects the relationship between income inequality and healthcare access. Our findings suggest that Internet access mitigates the negative impact of income inequality on healthcare access, which reinforces the important role of the Internet in shaping health outcomes. Third, this paper contributes to a better understanding of the factors associated with health inequality and healthcare access based on representative data. This paper conducts an empirical study using cross-country panel data covering a large number of developed and developing countries over a period of more than two decades. The rich and comprehensive data allow us to fully exploit the variations across countries and over time in the estimations. It provides a useful tool for future research investigating longstanding health disparities and the means to leverage new technology to narrow the gaps in public health.

The rest of the paper is organized as follows. Section 2 provides the literature review. Section 3 describes the methodology, including the data and sample, measurements of variables, and estimation methods. Section 4 presents the results, including descriptive statistics, baseline results, and robustness checks. Section 5 discusses the implications of and future directions for research. Finally, Section 6 concludes.

## Literature Review

This paper builds upon two strands of literature. The first strand of literature examines the impacts of Internet access on economic development and health outcomes. The second strand of literature focuses on health inequality, healthcare access, and their determinants and measurements.

The Internet is the global system of interconnected computer networks that has revolutionized communications and commercial modes by allowing information to be distributed and accessed effortlessly from anywhere (11). The Internet, which carries a wide variety of resources and services, also provides a powerful and general ability to support access to digital information by numerous applications (12). Over past decades, technological progress has yielded substantial performance improvements in networking and resulted in significantly declining unit costs of information processing (13). These performance improvements and the associated cost reductions have greatly accelerated the diffusion of Internet access since the 1990s, although considerable regional disparities remain (14, 15). Economic activities in the Internet age are increasingly interrelated due to complementarities among dense networks, online services and diverse applications (16). While national economies and large enterprises have reaped large benefits from the technological revolution, individual consumers and small businesses have been some of the major beneficiaries of the Internet's empowering influence (17, 18). In addition to economic development, the Internet is playing a vital role in influencing health outcomes (3, 4, 19, 20). The diffusion of the Internet has dramatically reduced informational frictions and given people unprecedented sources of health information (5, 21, 22). Access to health information via the Internet is redefining the roles of patients and medical practitioners since the flow of health information to patients is no longer controlled by physicians (23). It has been well documented that a remarkably large proportion of Internet users look online for information about health (24). Internet access is found to be positively correlated with the use of healthcare and thus health outcomes, providing evidence underlining the growing importance of the Internet as a valuable source of health related information (6, 25–27). Internet-based telehealth encompasses a wide range of physician-patient communication through online portals so that people are able to access medical services remotely and take better control of their healthcare (9, 10, 28). Moreover, eHealth and health informatics, with a broad definition covering healthcare practices supported by digital processes and Internet communications, improve the health, well-being, and economic functioning of society by promoting the efficient and effective use and analysis of information in the Internet era (29–32). Despite a number of health benefits provided by the Internet, it has also been documented in the literature that accessing health information on the Internet and the rapidly evolving digital environment could lead to the problems of healthcare misinformation, health-related fake news, and infodemic (33–35). These problems become more pronounced during the pandemic and could cause adverse health effects (36–38). In consideration of the fact that the Internet offers widespread access to health information and become an important resource to learn about health for users worldwide, the health benefits associated with Internet access still outweigh the potential drawbacks (39).

There is ample evidence in the literature that various social and economic factors, including age, education, gender, ethnicity, family status, employment status and income level, have important impacts on people's health (40–42). Health inequities are systematic differences in the health status of different population groups, which can be observed in many health outcomes such as life expectancy, self-assessed mental health, and mortality (2). These inequities can impose significant social and economic costs on both individuals and societies (1, 43). Public health practitioners, researchers, and policy-makers have long endeavored not only to improve overall health status but also to reduce health inequality through the right mix of public health policies (44). Increasing the use of health information technology, enhancing health literacy, and implementing health programs targeting socially disadvantaged groups are possible means of promoting health equity (45, 46). Several methods have been employed in the literature to measure the degree of health inequality. The concentration index approach, which quantifies the extent of socioeconomic-related inequality in a health indicator, has enjoyed increasing popularity in related studies (47). A major component of achieving universal health coverage is ensuring that people of different socioeconomic groups have access to quality healthcare (48). Social, economic, demographic, cultural, and geographic factors affect people's ability to access healthcare (49). Limited availability of health services is a barrier that reduces access to healthcare. For instance, physician shortages could lead to longer wait times and delayed care (50). The effective implementation of information technology in healthcare is part of the solution to this problem (51). Healthcare access and quality can be approximated by measuring mortality rates from causes that should not be fatal in the presence of effective and high-quality medical treatments (i.e., amenable mortality). Improving healthcare access is an important step toward reducing health inequality (52).

## Methodology

### Data and Sample

This paper conducts a two-tier empirical analysis using cross-country panel data. First, to examine the impact of Internet access on health inequality, we employ the WHO Health Equity Assessment Toolkit (HEAT), which enables users to calculate summary measures of health inequality using an existing database of disaggregated data (53, 54). HEAT contains disaggregated data from the WHO Health Equity Monitor database (2021 update). The data are based on reanalysis of Demographic and Health Surveys (DHS), Multiple Indicator Cluster Surveys (MICS) and Reproductive Health Surveys (RHS) conducted in various countries. The same methods of calculation for data analysis were applied across all surveys in to generate comparable estimates across countries and over time. Specifically, HEAT allows for the assessment of health inequality based on a wide range of health indicators (e.g., reproductive, maternal, and newborn health) along different dimensions (e.g., economic status, education, place of residence, subnational region, age, and sex). Note that the sample period ranges from 1993 to 2019. Given that the survey data were not available annually for all countries, the overall sample is an unbalanced panel dataset comprising 83 countries and 272 country-year observations.^1^

Second, to further scrutinize the social and economic determinants of access to healthcare, we utilize the Global Burden of Disease (GBD) database, which provides a comprehensive and systematic worldwide assessment of mortality and morbidity from major diseases, injuries, and risk factors (55, 56). The GBD provides an important tool for quantifying health loss from numerous diseases, injuries, and risk factors so that health systems can be improved, and disparities can be reduced. Measuring mortality rates due to causes that are considered amenable to healthcare is one way to characterize average levels of personal healthcare access and quality (57–59). Based on cause of death data and risk exposure data and estimates from the GBD 2016, the Healthcare Access and Quality (HAQ) Index is constructed for 194 countries from 1990 to 2016. The sample, which is an unbalanced panel dataset due to data availability, consists of 1156 country-year observations.^2^

### Measurements of Variables

#### Dependent Variable

To measure the degree of health inequality (*HealthInequality*), we employ the absolute concentration index (ACI), which is calculated based on the infant mortality rate (*IMR*) in HEAT. The IMR is defined as the number of deaths per 1,000 live births of children under 1 year of age and is considered to be an important measure of health condition (60–62). It is widely used as an indicator to quantify the level of health disparity (63–65). Specifically, we focus on health inequality associated with economic status, which is determined using a wealth index. Regarding economic status, within each country, the wealth index was divided into 10 equal subgroups, namely, wealth deciles, in which each group accounts for 10% of the population. Subgroups are ranked from the most-disadvantaged (i.e., poorest) to the most-advantaged subgroup (i.e., richest).

The ACI is calculated as:


(1) $$\begin{array}{r} ACI=\underset{j}{\sum }p_{j}\left(2x_{j}-1\right)y_{j} \end{array}$$

Where, *p*_*j*_ denotes the population share of subgroup *j*, *x*_*j*_ represents the relative rank of subgroup *j*, and *y*_*j*_ indicates the estimate for subgroup *j*. Note that, if there is no inequality, the ACI is equal to zero. Negative values indicate a concentration of the IMR among the poor subgroups, while positive values indicate a concentration of the IMR among the rich subgroups. The ACI characterizes the health inequality across population subgroups with different economic statuses.

To measure and evaluate healthcare accessibility (*HealthcareAccess*) across countries and over time, the HAQ Index is constructed on the basis of principal component analysis, providing an overall score of healthcare access and quality on a scale of 0–100 across locations from 1990 to 2016. The HAQ Index is sourced from the GBD 2016 results (66–68). Patterns of performance on the overall HAQ Index vary considerably across countries, with most countries in the highest decile located in Europe and almost all of the countries in the lowest decile clustered in sub-Saharan Africa. These substantial variations in the HAQ Index, both across countries and over time, allow us to examine the social and economic determinants of access to healthcare.

#### Key Independent Variable

The key independent variable of interest is the Internet access (*Internet*), that is, the individuals using the Internet as a proportion of the population. Internet access data are sourced from the World Development Indicators (WDI) developed by the World Bank. According to the definition of the indicator, Internet users are individuals who have used the Internet (from any location) through a computer, mobile phone, personal digital assistant, digital TV, etc. Access to the Internet can be provided via a fixed or mobile network.

Income inequality (*IncomeInequality*) is characterized by the Gini coefficient, which is a synthetic measure of statistical dispersion intended to represent the inequality within a nation. The Gini coefficient ranges from 0 (in the case of perfect equality) to 1 (a situation in which one person has all of the income and everyone else has none in an economy). A higher Gini coefficient indicates greater income inequality, with high-income individuals receiving much larger shares of the total income of the population. Previous studies have emphasized the relationship between income inequality and health outcomes (69–72). Following previous studies (73), income inequality data are collected from the Standardized World Inequality Database (SWIID).

#### Control Variables

The set of control variables includes gross domestic product (GDP), GDP per capita, trade liberalization, and government effectiveness, which are described in detail below.

(1) *GDP* is the monetary measure of all the final goods and services produced in a specific time period by a country. We use the logarithm of GDP to measure a nation's overall economic development. GDP data are in current U.S. dollars and are taken from the WDI.

(2) GDP per capita (*GDPPC*) is determined by dividing GDP by the population. We use the logarithm of GDP per capita to proxy the standard of living in a country. GDP per capita data are sourced from the WDI and measured in current U.S. dollars.

(3) Trade liberalization (*TL*) is the sum of exports and imports of goods and services measured as a share of GDP. We employ the measure of trade liberalization to characterize the degree to which countries are open to international trade. It has been documented in the literature that trade liberalization plays an important role in shaping health inequality and healthcare access (74–76). Trade liberalization data are collected from the WDI.

(4) Government effectiveness (*GE*) captures the perception of the quality of public service, the quality of civil service and the extent of its independence from political pressure, the quality of policy formulation and implementation, and the credibility of the government's commitment to these policies. The measure of government effectiveness is reported in the standard normal unit, with a mean of zero and a standard deviation of one and ranging from ~-2.5 to 2.5, with higher values corresponding to better governance. It has been shown in the literature that government effectiveness and institutional performance have important impacts on health outcomes (77–79). Government effectiveness data are sourced from the Worldwide Governance Indicators (WGI) (80).

Table 1 summarizes all of the variables used in this study, including variable names, measures, codes, and sources.

**Table 1:** Summary of variables.

**Variable**	**Measure**	**Code**	**Source**
**Panel A: The impact of Internet access on health inequality (1993–2019)**
Health inequality	The absolute concentration index calculated based on the infant mortality rate across different economic status groups in a country	HealthInequality	WHO
Health indicator	The average infant mortality rate in a country	IMR	WHO
**Panel B: The social and economic determinants of access to healthcare (1990–2016)**
Healthcare accessibility	The overall score of healthcare access and quality in a country	HealthcareAccess	GBD
Income inequality	GINI index, a synthetic measure of statistical dispersion to represent the inequality in a country	IncomeInequality	SWIID
**Panel C: Independent and control variables in both analyses**
Internet access	The individuals using the Internet as a proportion of the population	Internet	WDI
Gross domestic product	The logarithm of GDP in current U.S. dollars	GDP	WDI
Gross domestic product per capita	The logarithm of GDP per capita in current U.S. dollars	GDPPC	WDI
Trade liberalization	The ratio of the sum of exports and imports of goods and services to GDP	TL	WDI
Government effectiveness	Government effectiveness index from the Worldwide Governance Indicators	GE	WGI

### Estimation Methods

In the first step, to explore how Internet access affects health inequality, we estimate the following equation:


(2) $$HealthInequality_{it}=\alpha +\beta \times Internet_{it}+X_{it}^{\prime }\Gamma +D_{t}+\varepsilon_{it}$$

In Equation (2), *HealthInequality*_*it*_ represents the degree of health inequality of country *i* in year *t*, and *Internet*_*it*_ denotes the level of Internet access of country *i* in year *t*. The key coefficient of interest, β, captures the impact of Internet access on health inequality. Note that **X**_**it**_ is a vector of various control variables, and *D*_*t*_ indicates the time fixed effect. Following convention, α is the intercept and ε_*it*_ is the idiosyncratic disturbance term.

In the second step, to further examine the social and economic determinants of healthcare access, we adopt the following estimation:


(3) $$\begin{array}{l} HealthcareAccess_{it}=\alpha +\beta_{1}\times Internet_{it}+\beta_{2}\\ \;\times IncomeInequality_{it}+\beta_{3}\times Internet_{it}\\ \;\times IncomeInequality_{it}+X_{it}^{\prime }\Gamma \\ \;+D_{i}+D_{t}+\varepsilon_{it} \end{array}$$

In Equation (3), *HealthcareAccess*_*it*_ indicates the healthcare accessibility of country *i* in year *t*, *Internet*_*it*_ denotes the level of Internet access of country *i* in year *t*, and *IncomeInequality*_*it*_ represents the degree of income inequality of country *i* in year *t*. The main coefficients of interest, β_1_, β_2_, and β_3_, characterize the effects of Internet access, income inequality, and the interaction between Internet access and income inequality on access to healthcare, respectively. **X**_**it**_ is a vector of various control variables as in Equation (2). *D*_*i*_ denotes the country fixed effect and *D*_*t*_ indicates the time fixed effect. Last, α is the constant term and ε_*it*_ is the error term.

## Results

### Descriptive Statistics

Table 2 displays the descriptive statistics of all of the variables in the empirical estimations. As indicated in Table 2, there are large variations in health inequality, healthcare access and Internet access both across countries and over time, enabling us to systematically explore their relationships using estimation methods.

**Table 2:** Descriptive statistics.

**Variable Code**	**Observation**	**Mean**	**Standard deviation**	**Minimum**	**Maximum**
**Panel A: The impact of Internet access on health inequality (1993–2019, 83 countries)**					
HealthInequality	272	−5.230	3.792	−16.604	3.760
IMR	272	55.642	27.408	5.146	147.377
Internet	272	12.661	15.391	0	66.790
GDP	272	23.695	1.620	19.115	28.375
GDPPC	272	7.106	0.940	5.321	9.443
TL	272	65.944	31.336	1.378	194.351
GE	272	−0.631	0.460	−2.058	0.658
**Panel B: The social and economic determinants of access to healthcare (1990–2016, 194 countries)**					
HealthcareAccess	1,164	52.967	22.524	10.600	97.100
IncomeInequality	771	38.178	8.445	17.300	62.300
Internet	1,156	18.027	26.164	0	98.240
GDP	1,105	23.525	2.377	17.499	30.560
GDPPC	1,105	8.031	1.586	4.556	11.561
TL	1,055	83.962	51.348	0.021	583.314
GE	1,116	−0.054	0.987	−2.260	2.241

### Baseline Results

Table 3 displays the estimation results regarding the influence of the Internet on health inequality. As shown in Column (1) of Table 1, the coefficient of Internet access is positive and statistically significant at the 5% level. It is worth noting that the mean value of the ACI is −5.23, and the median value of the ACI is −4.68, implying a concentration of the IMR among the poor subgroups. A higher level of Internet access is associated with an increase in ACI and a decrease in health inequality. Therefore, in terms of health condition, the gap between the poor and the rich would be reduced if the Internet became more accessible. Column (2) of Table 1 indicates that the effect stemming from the Internet remain positive and significant after controlling for a wide variety of variables that influence health inequality.

**Table 3:** The impact of Internet access on health inequality (1993–2019).

**Model**	**HealthInequality**	
**Variables**	**(1)**	**(2)**
Internet	0.040^**^	0.036^**^
	(0.016)	(0.018)
GDP		−0.549^***^
		(0.193)
GDPPC		0.645^*^
		(0.360)
TL		0.012
		(0.011)
GE		−0.395
		(0.772)
constant	−5.735^***^	1.714
	(0.423)	(4.773)
Year fixed-effect	Yes	Yes
Observation	272	272
*R* ^2^	0.217	0.292
Adjusted *R*^2^	0.199	0.265

*Standard errors in parentheses*.

* *p < 0.10*.

** *p < 0.05*.

*** *p < 0.01*.

Table 4 reports the regression results regarding the impact of Internet access on the IMR. The identification essentially replaces the dependent variable in Equation (2) with the IMR of country *i* in year *t* (i.e., *IMR*_*it*_), allowing us to further explore the influence of the Internet on the overall IMR, in addition to health inequality. Column (1) of Table 2 shows that the coefficient of Internet access is negative and statistically significant at the 1% level. The magnitude of the coefficient implies that a 10% increase in the level of Internet access is related to a decrease of approximately 0.11 deaths per 1,000 live births. When including a full set of control variables in the estimation, Column (2) of Table 2 demonstrates that, all else being equal, a higher level of Internet access is related to a decline in the overall IMR. In addition, as expected, variables such as GDP, GDP per capita, trade liberalization, and government effectiveness tend to decrease the average level of IMR.

**Table 4:** The impact of Internet access on the IMR (1993–2019).

**Model**	**IMR**	
**Variables**	**(1)**	**(2)**
Internet	−1.087^***^	−0.219^*^
	(0.102)	(0.125)
GDP		−1.490
		(1.512)
GDPPC		−12.150^***^
		(2.557)
TL		−0.110^*^
		(0.064)
GE		−9.695^***^
		(3.621)
constant	69.410^***^	181.213^***^
	(2.308)	(36.667)
Year fixed-effect	Yes	Yes
Observation	272	272
*R* ^2^	0.467	0.622
Adjusted *R*^2^	0.408	0.573

*Standard errors in parentheses*.

* *p < 0.10*.

^**^p < 0.05.

*** *p < 0.01*.

Table 5 shows the estimation results of Equation (3), focusing on the social and economic determinants of healthcare access. Columns (1) and (2) of Table 3 include only the factors of Internet access and income inequality, respectively. It is found that an improvement in Internet access significantly facilitates healthcare access, while an increase in income inequality significantly impedes access to healthcare. Column (3) of Table 3 further incorporates various control variables and confirms the similar effects of Internet access and income inequality on healthcare access. Finally, Column (4) of Table 3 includes the interaction term between Internet access and income inequality. The coefficient of the interaction term is positive and statistically significant, implying that Internet access mitigates the negative impact of income inequality on healthcare access.

**Table 5:** The social and economic determinants of access to healthcare (1990–2016).

**Model**	**HealthcareAccess**			
**Variables**	**(1)**	**(2)**	**(3)**	**(4)**
Internet	0.028^***^		0.021^***^	−0.023
	(0.006)		(0.008)	(0.021)
IncomeInequality		−0.165^***^	−0.160^***^	−0.149^***^
		(0.055)	(0.053)	(0.053)
GDP			5.462^***^	5.078^***^
			(1.117)	(1.125)
GDPPC			−1.869	−1.577
			(1.146)	(1.148)
TL			0.013^**^	0.015^***^
			(0.005)	(0.005)
GE			1.671^***^	1.697^***^
			(0.474)	(0.472)
Internet × IncomeInequality				0.001^**^
				(0.001)
constant	52.519^***^	62.916^***^	−55.398^***^	−49.183^**^
	(0.142)	(2.104)	(19.154)	(19.265)
Country fixed-effect	Yes	Yes	Yes	Yes
Year fixed-effect	Yes	Yes	Yes	Yes
Observation	1,156	763	717	717
*R* ^2^	0.988	0.991	0.993	0.993
Adjusted *R*^2^	0.985	0.988	0.991	0.991

*Standard errors in parentheses*.

^*^p < 0.10.

** *p < 0.05*.

*** *p < 0.01*.

### Granger Causality Test

In our study, the relationships among the Internet, health inequality and healthcare access have been empirically identified. However, it is important to determine the causality among these variables to further establish the cause-effect links. Following previous studies (81–83), we conduct dynamic panel Granger causality tests to analyze the causal relationship among the key variables. The results of the Granger causality tests are presented in Table 6. As shown in Panel A of Table 6, only the F-statistic of *Internet* to *HealthInequality* is significant at 5%, indicating that the Internet is an important and robust cause of health inequality, but health inequality does not cause the diffusion of Internet in the reverse direction. As seen from Panel B of Table 6, the bidirectional causality between *Internet* and *HealthcareAccess* is significant at 1%. To this end, policies aimed at boosting Internet development will eventually improve healthcare access in the long run. Last, the F-statistic of *IncomeInequality* to *HealthcareAccess* is significant at 5%, implying that income inequality is an important and robust explanatory variable for healthcare access.

**Table 6:** Panel causality tests.

	**F-statistics**	***p*-value**	**Null hypothesis for the tests**
**Panel A: The impact of Internet access on health inequality (1993–2019)**			
Internet → HealthInequality	4.458	0.035	Internet does not Granger-cause HealthInequality.
HealthInequality → Internet	0.347	0.556	HealthInequality does not Granger-cause Internet.
**Panel B: The social and economic determinants of access to healthcare (1990–2016)**			
Internet → HealthcareAccess	522.965	0.000	Internet does not Granger-cause HealthcareAccess.
HealthcareAccess → Internet	126.973	0.000	HealthcareAccess does not Granger-cause Internet.
IncomeInequality → HealthcareAccess	6.053	0.014	IncomeInequality does not Granger-cause HealthcareAccess.
HealthcareAccess → IncomeInequality	0.361	0.548	HealthcareAccess does not Granger-cause IncomeInequality.

### Robustness Checks

To validate the empirical findings in Tables 3–5, we further conduct a wide set of robustness checks using alternative estimations, different samples, alternative measures, and subsamples.

Regarding the impact of Internet access on health inequality, Table 7 shows the results using pooled OLS estimations. The pooled OLS estimation results, in which the data on different units are pooled together with no assumptions about individual differences, are consistent with the fixed-effect estimates in Table 3. This pattern further affirms that access to the Internet significantly reduces health inequality.

**Table 7:** Robustness checks using alternative estimations.

**Model**	**(1)**	**(2)**
**Sample**	**WHO Sample**	
**Variables**	**HealthInequality**	
Internet	0.089^***^	0.077^***^
	(0.014)	(0.018)
GDP		−0.450^***^
		(0.171)
GDPPC		0.774^**^
		(0.339)
TL		0.017^**^
		(0.008)
GE		−1.402^***^
		(0.477)
constant	−6.363^***^	−3.064
	(0.278)	(3.897)
Year fixed-effect	No	No
Observation	272	272
*R* ^2^	0.467	0.622
Adjusted *R*^2^	0.408	0.573

*Robustness checks related to Table 3 about the impact of Internet access on health inequality*.

*Standard errors in parentheses*.

^*^p < 0.10.

** *p < 0.05*.

*** *p < 0.01*.

With respect to the impact of Internet access on the IMR, Table 8 displays the results using different samples with more observations. Note that the WDI includes more extensive data on the IMR, as compared to HEAT. The results based on larger samples are qualitatively similar to the results presented in Table 4. This outcome implies that increased Internet access is associated with a decrease of the overall IMR.

**Table 8:** Robustness checks using different samples.

**Model**	**(1)**	**(2)**
**Sample**	**WDI Sample**	
**Variables**	**IMR**	
Internet	−0.706^***^	−0.063^**^
	(0.013)	(0.027)
GDP		0.156
		(0.167)
GDPPC		−10.214^***^
		(0.432)
TL		−0.001
		(0.007)
GE		−4.371^***^
		(0.648)
constant	50.581^***^	111.670^***^
	(0.548)	(4.692)
Year fixed-effect	Yes	Yes
Observation	2,818	2,818
*R* ^2^	0.505	0.624
Adjusted *R*^2^	0.502	0.622

*Robustness checks related to Table 4 about the impact of Internet access on the IMR*.

*Standard errors in parentheses*.

^*^p < 0.10.

** *p < 0.05*.

*** *p < 0.01*.

With regard to the social and economic determinants of healthcare access, Tables 9, 10 report the results using alternative measures of Internet access and subsamples based on the median value of Gini coefficient, respectively. Since the key explanatory variable of interest is Internet access, we utilize two alternative measures, namely the number of Internet servers per million people and the number of broadband Internet subscribers per hundred people, both of which are sourced from the WDI. Columns (1) and (2) of Table 9 confirm that better access to the Internet significantly improves the healthcare access. The results are robust to the alternative measures of Internet access. In addition, to justify the moderating effect of Internet access, we categorize the total sample into two subsamples of equal size based on the degree of income inequality. Columns (1) and (2) of Table 10 report the estimation results using subsamples that are greater and less than the median value of the Gini coefficient (i.e., 38.2), respectively. The positive and significant impact of the Internet on healthcare access exists in countries that are more unequal in terms of income distribution (i.e., higher Gini coefficient). The analogous positive and significant effect does not exist in countries with a lower degree of income inequality (i.e., lower Gini coefficient). Taken together, the subsample analysis suggests that access to the Internet tends to bridge the gap in healthcare access between the poor and the rich in highly unequal societies. This finding provides evidence of the moderating role of Internet access, consistent with the baseline results in Table 5.

**Table 9:** Robustness checks using alternative measures.

	**(1)**	**(2)**
**Sample**	**Full Sample**	
**Variables**	**HealthcareAccess**	
Internet_server	0.714^***^	
	(0.179)	
Internet_broadband		0.306^***^
		(0.101)
IncomeInequality	−0.197	−0.020
	(0.130)	(0.068)
GDP	3.632	1.269
	(2.889)	(1.534)
GDPPC	−1.977	0.298
	(3.048)	(1.630)
TL	−0.012	−0.001
	(0.008)	(0.006)
GE	0.073	1.425^***^
	(0.842)	(0.537)
constant	4.232	32.210
	(48.236)	(25.934)
Country fixed-effect	Yes	Yes
Year fixed-effect	Yes	Yes
Observation	144	349
*R* ^2^	0.999	0.997
Adjusted *R*^2^	0.997	0.996

*Robustness checks related to Table 5 about the social and economic determinants of access to healthcare; b. Standard errors in parentheses*.

^*^p < 0.10.

^**^p < 0.05.

*** *p < 0.01*.

*The WDI reports the worldwide data on the number of secure Internet servers since 2010, and the number of fixed broadband Internet subscribers since 2000. Thus, the sample sizes are smaller than the estimations using Internet access as the key independent variable in Table 5*.

**Table 10:** Robustness checks using subsamples.

	**(1)**	**(2)**
**Sample**	**High Gini Group**	**Low Gini Group**
**Variables**	**HealthcareAccess**	
Internet	0.075^***^	−0.002
	(0.017)	(0.009)
GDP	2.483	6.154^***^
	(1.808)	(1.482)
GDPPC	1.640	−2.909*
	(1.906)	(1.481)
TL	0.047^***^	0.005
	(0.011)	(0.005)
GE	2.714^***^	1.775^***^
	(0.746)	(0.601)
constant	−30.341	−57.874**
	(29.847)	(24.985)
Country fixed-effect	Yes	Yes
Year fixed-effect	Yes	Yes
Observation	361	356
*R* ^2^	0.986	0.993
Adjusted *R*^2^	0.981	0.991

*Robustness checks related to Table 5 about the moderating effect of Internet access*.

*Standard errors in parentheses*.

^*^p < 0.10.

^**^p < 0.05.

*** *p < 0.01*.

*The high Gini group is defined as the subsample of observations that are greater than the median value of the Gini coefficient (i.e., 38.2), while the low Gini group is defined as the subsample of observations that are less than the median value of the Gini coefficient*.

## Discussion and Implications

### Policy Implications

This paper sheds empirical light on the relationship between access to the Internet and major health outcomes, which could provide several important implications for policy-makers. First, health inequality refers to the unjust and avoidable differences in health across the population and between different population groups. These widespread differences have detrimental effects on people's living conditions and overall health status (1, 2, 43). In particular, health inequality affects people from disadvantaged groups most severely and goes against the principle of social justice. Reducing health inequalities within and between countries becomes a social, economic and ethical imperative for policy-makers. This paper shows that access to the Internet plays a pivotal role in reducing health inequality across the social gradient. Therefore, governments should increase investments in digital infrastructure and promote the continuous development of the Internet and ICTs, as a means of redressing longstanding inequality in health.

Second, ensuring access to quality healthcare is a crucial component of achieving universal health coverage. The main determinants of healthcare access are the social and economic conditions in which people live that influence health outcomes throughout life (40, 45, 49, 65). By carefully examining the social and economic determinants of healthcare access, this paper points to the moderating role of Internet access on the relationship between income inequality and healthcare access. Since Internet connectivity not only significantly improves healthcare access but also mitigates the negative effect of income inequality, policy-makers aiming to promote access to healthcare should consider the value of the Internet as an important tool to improve healthcare. Governments ought to increase the network coverage and lower barriers to accessing the Internet, especially for disadvantaged groups, which generally have worse health outcomes and suffer from a lack of access to quality healthcare.

Third, given the importance of the Internet in shaping health outcomes through the distributional and aggregate effects (3, 8, 25), ensuring that health outcomes are equitable across different population groups will be as crucial as utilizing new technology in healthcare to improve these outcomes. Policy-makers should pay close attention to the digital divide, which is the uneven distribution in access to, use of, or impact of the Internet and ICTs between different population groups. These distinct groups might be defined based on social, geographical, ethnic, or economic criteria. Thus, bridging the digital divide could reduce disparities in health outcomes and reap the benefits of improving healthcare access (84). Meanwhile, given a vast quantity of inaccurate information online, healthcare misinformation, health-related fake news, and particularly infodemic during a disease outbreak could lead to the spread of ineffective and even harmful public health measures (36–38). It is of vital importance to build up necessary skills needed by public health practitioners to deliver fast, efficient, and cost-effective responses to the challenges of misinformation and health-related fake news. Policy-makers responsible for health communication strategies and social media policies can adopt preventive measures to cope with infodemic during the pandemic (85).

### Limitations and Future Research Directions

This study has made considerable contributions to the literature, but there are some limitations to be addressed in future research. First, since the digital economy has developed rapidly over the last few decades, a detailed study of the impact of the digital divide on disparities in healthcare is left for future research. Second, due to data availability, this study could not incorporate the COVID pandemic into the analysis. Future research could further explore the relationship between inequality and healthcare during the COVID pandemic period with updated data. As misinformation concerning health tends to have serious consequences with regard to health risks and outcomes, it is worth examining how people interact with healthcare misinformation online during the COVID pandemic. Exploring practical ways to leverage health communication strategies via the Internet to overcome COVID infodemic deserves further in-depth study. Finally, since this study focuses on a cross-country analysis at the macro-level, it would be interesting to investigate several micro-level (i.e., personal characteristics and/or family characteristics) determinants of income-related health inequalities in the digital era.

## Conclusion

The Internet has profoundly changed the way in which health information is shared and accessed, which has evolved with the ever-changing needs of both physicians and patients. Health information on the Internet significantly increases people's knowledge of, engagement in, and competence with health decision-making strategies. The Internet has been increasingly used for health-related purposes in the contemporary age and has major implications for public health, including health inequality and healthcare access. Despite the importance of this topic to the field of public health, few comprehensive studies have been conducted to explore their relationships. This paper contributes to the literature by systematically examining the impacts of the Internet on health inequality and healthcare access based on a cross-country study. The major findings of this paper are twofold. First, Internet access significantly reduces health inequality across different income groups and increases the average health condition. Second, access to the Internet significantly facilitates healthcare access and mitigates the negative impact of income inequality on healthcare access. More importantly, the results persist across a wide variety of robustness checks.

## Data Availability Statement

Publicly available datasets were analyzed in this study. This data can be found here: 1. WHO Health Equity Assessment Toolkit: https://www.who.int/data/health-equity/assessment_toolkit. 2. Global Burden of Disease database: https://ghdx.healthdata.org/gbd-2017. 3. World Bank Database: https://databank.worldbank.org/home.aspx. 4. Standardized World Inequality Database: https://www.wider.unu.edu/project/world-income-inequality-database-wiid.

## Author Contributions

JY: conceptualization, methodology, validation, and writing—original draft. SM: conceptualization, methodology, visualization, and writing—review and editing. Both authors contributed to the article and approved the submitted version.

## Funding

This work was supported by the Fundamental Research Funds for the Central Universities and the Humanities and Social Science Fund of Ministry of Education of China (Grant No. 20YJC790099).

## Conflict of Interest

The authors declare that the research was conducted in the absence of any commercial or financial relationships that could be construed as a potential conflict of interest.

## Publisher's Note

All claims expressed in this article are solely those of the authors and do not necessarily represent those of their affiliated organizations, or those of the publisher, the editors and the reviewers. Any product that may be evaluated in this article, or claim that may be made by its manufacturer, is not guaranteed or endorsed by the publisher.

## Footnotes

^1^The list of countries and territories is presented in the Supplementary Table S1.

^2^The list of countries and territories is presented in the Supplementary Table S2.
