# Supplementary material for: Impacts of the Internet on Health Inequality and Healthcare Access: A Cross-Country Study
Source: Front Public Health. 2022 Jun 9;10:935608. doi: 10.3389/fpubh.2022.935608 (PMC9218541; doi:10.3389/fpubh.2022.935608)
Supplement: Supplementary file 1 [file Data_Sheet_1.PDF]

Supplementary Material
"Impacts of the Internet on Health Inequality and Healthcare Access: A Cross-country Study"

Table S1. The list of countries and territories in the WHO Health Equity Assessment Toolkit database

Albania, Algeria, Angola, Armenia, Azerbaijan, Bangladesh, Benin, Bolivia, Brazil, Burkina Faso, Burma, Burundi, Cambodia, Cameroon, Central African Republic, Chad, Cote d'Ivoire, Colombia, Comoros, Democratic Republic of Congo, Dominican Republic, Egypt, El Salvador, Eswatini, Ethiopia, Gabon, Gambia, Ghana, Guatemala, Guinea, Guinea-Bissau, Guyana, Haiti, Honduras, Indonesia, India, Iraq, Jordan, Kazakhstan, Kenya, Kiribati, Kyrgyz Republic, Laos, Lesotho, Madagascar, Maldives, Mali, Mauritania, Moldova, Mongolia, Morocco, Mozambique, Namibia, Nepal, Nicaragua, Niger, Nigeria, Pakistan, Papua New Guinea, Paraguay, Peru, Philippines, Republic of Congo, Rwanda, Senegal, Sierra Leone, South Africa, South Sudan, Sudan, Suriname, Tajikistan, Tanzania, Timor-Leste, Togo, Tunisia, Turkey, Turkmenistan, Uganda, Uzbekistan, Vietnam, Yemen, Zambia, Zimbabwe.

Table S2. The list of countries and territories in the Global Burden of Disease database

Afghanistan, Albania, Algeria, American Samoa, Andorra, Angola, Antigua and Barbuda, Argentina, Armenia, Australia, Austria, Azerbaijan, Bahamas, Bahrain, Bangladesh, Barbados, Belarus, Belgium, Belize, Benin, Bermuda, Bhutan, Bolivia, Bosnia and Herzegovina, Botswana, Brazil, Brunei Darussalam, Bulgaria, Burkina Faso, Burundi, Cabo Verde, Cambodia, Cameroon, Canada, Central African Republic, Chad, Chile, China, Colombia, Comoros, Costa Rica, Cote d'Ivoire, Croatia, Cuba, Cyprus, Czech Republic, Democratic People's Republic of Korea, Democratic Republic of Congo, Denmark, Djibouti, Dominica, Dominican Republic, Ecuador, Egypt, El Salvador, Equatorial Guinea, Eritrea, Estonia, Eswatini, Ethiopia, Federated States of Micronesia, Fiji, Finland, France, Gabon, Gambia, Georgia, Germany, Ghana, Greece, Greenland, Grenada, Guam, Guatemala, Guinea, Guinea-Bissau, Guyana, Haiti, Honduras, Hungary, Iceland, India, Indonesia, Iraq, Ireland, Islamic Republic of Iran, Israel, Italy, Jamaica, Japan, Jordan, Kazakhstan, Kenya, Kiribati, Kuwait, Kyrgyz Republic, Lao PDR, Latvia, Lebanon, Lesotho, Liberia, Libya, Lithuania, Luxembourg, Madagascar, Malawi, Malaysia, Maldives, Mali, Malta, Marshall Islands, Mauritania, Mauritius, Mexico, Moldova, Mongolia, Montenegro, Morocco, Mozambique, Myanmar, Namibia, Nepal, Netherlands, New Zealand, Nicaragua, Niger, Nigeria, North Macedonia, Northern Mariana Islands, Norway, Oman, Pakistan, Panama, Papua New Guinea, Paraguay, Peru, Philippines, Poland, Portugal, Puerto Rico, Qatar, Republic of Congo, Republic of Korea, Romania, Russian Federation, Rwanda, Samoa, Sao Tome and Principe, Saudi Arabia, Senegal, Serbia, Seychelles, Sierra Leone, Singapore, Slovak Republic, Slovenia, Solomon Islands, Somalia, South Africa, South Sudan, Spain, Sri Lanka, St. Lucia, St. Vincent and the Grenadines, Sudan, Suriname, Sweden, Switzerland, Syrian Arab Republic, Tajikistan, Tanzania, Thailand, Timor-Leste, Togo, Tonga, Trinidad and Tobago, Tunisia, Turkey, Turkmenistan, Uganda, Ukraine, United Arab Emirates, United Kingdom, United States, Uruguay, Uzbekistan, Vanuatu, Venezuela Vietnam, Virgin Islands, West Bank and Gaza, Yemen Republic, Zambia, Zimbabwe.
